# Fairness-Aware Machine Learning for Predicting Recovery and Dropout in NHS Talking Therapies

**DOI:** 10.1192/bjo.2026.11126

**Published:** 2026-06-30

**Authors:** Dominic Shipman, Anastasia Ushakova

**Affiliations:** 1 Lancaster Medical School, Lancaster, United Kingdom; 2 Lancashire and South Cumbria NHS Foundation Trust, Preston, United Kingdom

## Abstract

**Aims:**

Depression represents a leading cause of disability in England, with NHS Talking Therapies providing evidence-based psychological treatments to over 1.8 million people annually. Despite overall effectiveness, persistent disparities in recovery and engagement exist across demographic groups, particularly by age and ethnicity. Predictive modelling offers an opportunity to identify patients at risk of dropout or non-recovery, but few studies have evaluated such models for fairness.

This study aimed to develop and evaluate fairness-aware machine learning models to predict recovery and dropout in NHS Talking Therapies using early-treatment clinical data. In addition to assessing predictive performance, the study quantified algorithmic fairness across demographic groups, focusing on disparities in true positive rates by age and ethnicity. We hypothesised that integrating fairness constraints into model training would reduce demographic disparities while preserving predictive performance, thereby supporting more equitable and targeted service delivery.

**Methods:**

This study analysed routinely collected clinical data from 33,341 patients discharged from an NHS Foundation Trust in North-West England between January 2024 andJuly 2025. Outcomes included dropout after assessment, dropout before session four, and recovery following at least one therapy session. Predictor variables comprised demographic characteristics, referral pathway, and item-level Patient Health Questionnaire-9 (PHQ-9) responses, including early symptom change. Baseline models included L2-regularised logistic regression and Extreme Gradient Boosting (XGBoost). Performance was evaluated using the Area Under the Receiver Operating Characteristic Curve (AUROC), accuracy, and Equal Opportunity (parity in true positive rate (TPR)) across age, gender, and ethnicity. A custom fairness-aware XGBoost model was developed using a dynamic dual-ascent regularisation controller to reduce subgroup disparities during training.

**Results:**

Baseline models showed moderate discrimination (AUROC 0.65 for dropout after assessment; 0.75 for recovery), with logistic regression performing similarly to XGBoost. However, substantial disparities were observed, with TPR gaps exceeding 0.40 across age groups and 0.30 across ethnic groups. The fairness-aware XGBoost model maintained discrimination (AUROC 0.64–0.74) while markedly reducing subgroup disparities, achieving up to an 84% reduction in age-related gaps, approximately 50% in gender, and 40% in ethnicity, with only a minimal reduction in overall accuracy.

**Conclusion:**

These findings demonstrate that fairness-aware optimisation can mitigate demographic disparities in predictive modelling for mental health services while preserving predictive performance. To our knowledge, this represents the first application of fairness-constrained machine learning in NHS Talking Therapies. Such approaches could support more equitable, data-driven care pathways when implemented as clinician decision-support tools with ongoing fairness monitoring.

